# A 33-Year-Old Man with Gynaecomastia and Galactorrhea as the First Symptoms of Graves Hyperthyroidism

**DOI:** 10.1155/2016/1946824

**Published:** 2016-12-01

**Authors:** Somdul Khoohaphatthanakul, Apiradee Sriwijitkamol

**Affiliations:** ^1^Danchang Hospital, Suphan Buri, Thailand; ^2^Division of Endocrinology and Metabolism, Department of Medicine, Faculty of Medicine Siriraj Hospital, Mahidol University, Bangkok, Thailand

## Abstract

Graves' hyperthyroidism has a various number of well-recognized manifestations. Galactorrhea is a rare manifestation in this disease. We describe a 33-year-old man who presented with the symptoms of hyperthyroidism, gynaecomastia, and galactorrhea for 2 months. Physical examination revealed goitre, gynaecomastia, and galactorrhea, bilaterally. Laboratory investigations demonstrated high free thyroxine with suppressed thyroid-stimulating hormone level together with elevated anti-TSH receptor; therefore, the diagnosis of Graves' disease was confirmed. Other investigations to elucidate the etiology of galactorrhea were normal, so the galactorrhea was hypothesized to be caused by Graves' disease. The gynaecomastia and galactorrhea resolved with the successful treatment of hyperthyroidism. Although the galactorrhea is extremely rare in thyrotoxicosis male patients, to the best of our knowledge, this is the third case which reported gynaecomastia and galactorrhea in male patient who presented with thyrotoxicosis.

## 1. Introduction

Thyrotoxicosis is one of the possible causes of gynaecomastia and galactorrhea. The prevalence of gynaecomastia in thyrotoxicosis patients from two series [1, 2] has been reported as high as 40%. In contrast, galactorrhea has rarely been reported in Graves' disease and mostly reported in women [3]. Here, we describe a male patient who presented with symptoms and signs of thyrotoxicosis together with gynaecomastia and galactorrhea.

## 2. Case Presentation

**Figure 1:**
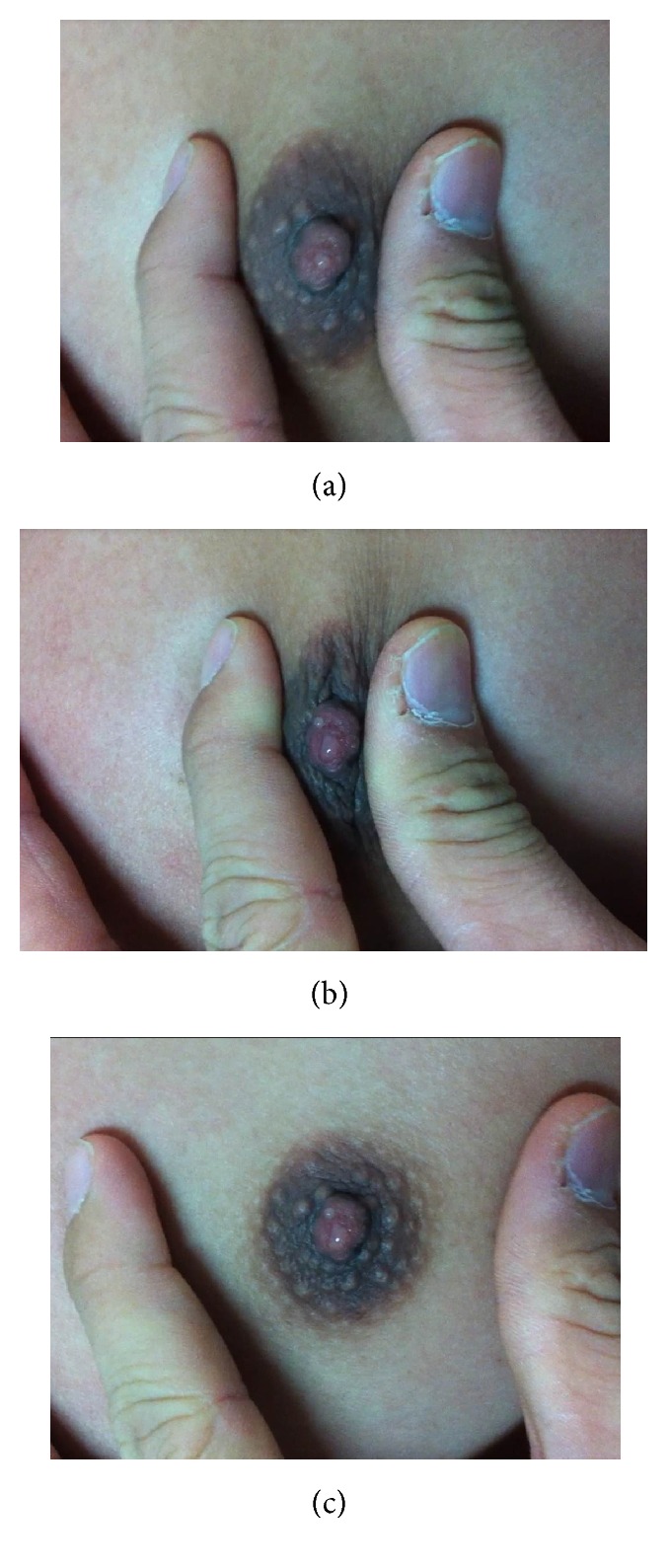
Galactorrhea. The pictures showed milky discharge from right breast, before (a), during (b), and after (c) gentle manipulation of the nipple.

A 33-year-old Thai man presented to the outpatient clinic in January 2015 with symptoms of hyperthyroidism, including lost of 3 kg body weight and hand tremor, during the period of 2 months. Interestingly, during the same time, he also noticed the enlargement of his breasts and the secretion from his nipples, bilaterally. He denied a history of erectile dysfunction, decreased libido, breast manipulation, drug ingestion, or use of medications including oral contraceptive pills and other medications. Examination revealed tachycardia, with the heartbeat of 106 bpm, moist skin, and onycholysis. The thyroid gland was soft and diffusely enlarged, with the weight of 30 grams, without nodule or thyroid bruit. Eye examination showed bilateral exophthalmos and lid retraction. Bilateral gynaecomastia with milky discharge from nipple on squeezing was seen (Figure 1).

His secondary sex characteristics and visual fields were normal. His laboratory investigations demonstrated high free thyroxine with suppressed thyroid-stimulating hormone level in company with highly positive anti-TSH receptor antibody. To investigate the causes of galactorrhea, plasma prolactin and creatinine level were measured and the results were normal, as shown in Table 1.

Based on symptoms, signs, and laboratory results, the diagnosis of Graves' disease was confirmed; therefore, 10 mg of methimazole twice a day was prescribed. He had gone through the conventional assessment, but the etiology of the galactorrhea was not revealed. The signs and symptoms of thyrotoxicosis improved together with normal free thyroxin (free T4 0.73, reference range 0.70–1.48 ng/mL) but thyroid-stimulating hormone level remains suppressed (TSH 0.01, reference range 0.35–4.94 mIU/L); in parallel with the disappearance of the galactorrhea after treatment with methimazole for 3 months, the gynaecomastia disappeared after treatment for 5 months.

## 3. Discussion

Thyrotoxicosis is one of the well-recognized possible causes of gynaecomastia and galactorrhea. The prevalence of gynaecomastia in male patients with thyrotoxicosis has been reported to be 10–40% [4]. The prevalence of gynaecomastia is wide variable percentage because of differences in ethnic group and in the criteria of diagnosis used in each report [4]. The reported frequency of galactorrhea in women with thyrotoxicosis is also variable from 1 to 80% [3], partly depending on lack of physical examination and differences in the criteria for diagnosis of galactorrhea. In contrast to women, galactorrhea is rare in men with the reported prevalence of 5.5% from 235 patients who presented with galactorrhea from any causes [5]. The galactorrhea is extremely rare in thyrotoxicosis male patients; to the best of our knowledge, this is the third case which reported gynaecomastia and galactorrhea in male patient who presented with thyrotoxicosis [6, 7]. Similar to our case, the gynaecomastia and galactorrhea disappeared after successful treatment of thyrotoxicosis [6, 7].

The pathophysiologic mechanisms responsible for a gynaecomastia with/without galactorrhea in thyrotoxicosis are a result of the increase in the physiologically active estrogen to androgen ratio and are not related to changes in prolactin secretion [3]. These conditions have been reported in both Graves hyperthyroidism and toxic multinodular goitre; therefore, the pathophysiology responsible for these conditions is linked to thyroid hormone, not to autoantibody [3, 8]. The mechanism by which thyrotoxicosis associated with changes in estrogen-testosterone ratio can be elucidated into 3 difference mechanisms. First of all, an increase in sex hormone binding globulin (SHBG) resulted in a decrease of physiologically active free testosterone [9]. SHBG is increased in thyrotoxicosis patients as a result of hepatic stimulation by thyroid hormones [3, 9]. Second of all, an elevated circulating estrogen level is caused by enhanced production rate of the estrogen and increment of peripheral conversion of androgen to estrogen [10, 11]. The last explanation is an increased level of androstenediol including 5-androsten-3*β*, 17~*β*-diol, and androstenediol 3-sulfate [8], which are active metabolites of dehydroepiandrosterone (DHEA) and DHEA sulfate, respectively. The estrogenic potential of androstenediol is only approximately 1% that of estradiol; however, the serum levels of androstenediol 3-sulfate are about 10,000-fold higher than that of estradiol [8]. As a result of these alterations, changes in the hormonal milieu (estrogen-testosterone ratio) play a fundamental role in the pathogenesis of gynaecomastia and galactorrhea in men with thyrotoxicosis. The limitation of our case was an absence of sex hormone level including estradiol, testosterone, DHEA, DHEA sulfate, LH, and FSH. However, we demonstrated the improvement of thyrotoxicosis associated with the improvement of these conditions; therefore, this could reasonably demonstrate the causal relationship between thyrotoxicosis and gynaecomastia/galactorrhea in our patient.

## Figures and Tables

**Table 1:** Laboratory investigation.

Laboratory investigation	Result	Reference range
Free T4 (ng/mL)	3.26	0.70–1.48
TSH (mIU/L)	<0.01	0.35–4.94
Prolactin (*μ*g/L)	10.54	3.46–19.40
Creatinine (mg/dL)	1	0.60–1.20
Anti-TSH receptor antibodies (IU/L)	27.98	0.00–1.75

Free T4: free thyroxine; TSH: thyroid-stimulating hormone.

## Abbreviations

TSH: Thyroid-stimulating hormone
T4: Thyroxin
SHBG: Sex hormone binding globulin
DHEA: Dehydroepiandrosterone
LH: Luteinizing hormone
FSH: Follicle stimulating hormone.
